# HSSAM-Net: hyper-scale shifted aggregation network for precise colorectal polyp segmentation in endoscopic images

**DOI:** 10.1038/s41598-025-21954-y

**Published:** 2025-10-31

**Authors:** Qing Feng, Shahzad Ahmed, Yueming Zhang, Lan He, Muhammad Yaqub

**Affiliations:** 1 School of Biomedical Sciences, Hunan University, Changsha, 410019 Hunan China; 2 Faculty of Information Technology, Beijing University of Technology, Beijing, China; 3 Department of General Surgery, The Third Hospital of Changsha, Changsha, 410082 Hunan China

**Keywords:** Colorectal cancer, Polyp segmentation, Medical image analysis, Attention mechanisms, Deep learning, Cancer, Image processing, Machine learning

## Abstract

Colorectal cancer remains a leading cause of cancer-related mortality worldwide, emphasizing the importance of early detection through accurate polyp identification. However, colonoscopy relies heavily on precise polyp segmentation in endoscopic images, yet this task remains challenging due to morphological variability, low contrast, and imaging artifacts. In this study, we propose HSSAM-Net, a lightweight deep learning framework that integrates a Hyper-Scale Shifted Aggregation Module to capture multi-scale contextual information while preserving fine-grained details, Progressive Reuse Attention mechanism that strengthens feature propagation across the encoder-decoder pathway, and Max-Diagonal Pooling/Unpooling (MaxDP/MaxDUP) a novel dual-branch sampling scheme to improve texture representation, feature alignment to enhance feature aggregation, context learning, and boundary refinement. The proposed model is evaluated on five benchmark datasets (Kvasir, CVC-ClinicDB, ETIS, CVC-300, EndoCV2020). Experimental results show that HSSAM-Net consistently outperforms state-of-the-art methods across benchmark datasets, HSSAM-Net consistently achieves state-of-the-art accuracy (Dice: 0.949–0.952, mIoU: 0.924–0.930), while maintaining real-time efficiency at 24.1 FPS with only 0.9 M parameters. Furthermore, an analysis of trainable parameters and inference speed confirms its suitability for real-time clinical applications. Our findings demonstrate that HSSAM-Net achieves a favorable trade-off between accuracy and efficiency, advancing the development of practical and reliable computer-aided colonoscopy systems.

**Supplementary Information:**

The online version contains supplementary material available at 10.1038/s41598-025-21954-y.

## Introduction

**Fig. 1:**
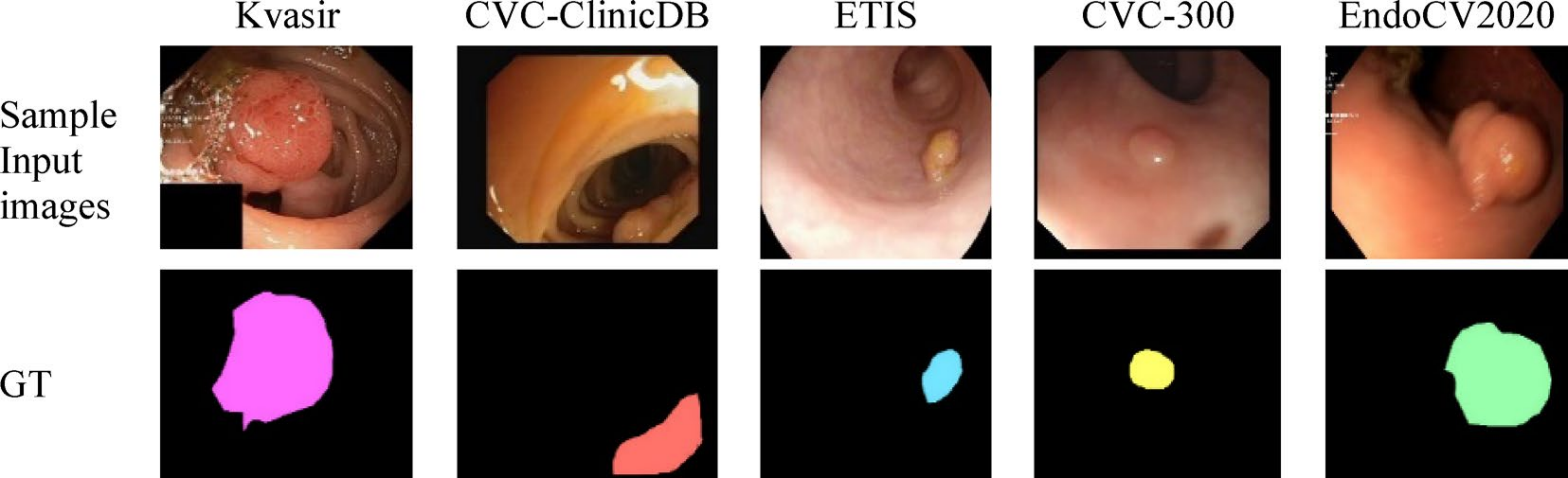
Sample input images and their corresponding ground truth masks from all five datasets used in this study for polyp segmentation.

CRC is one of the most prevalent malignancies worldwide, representing a major global health burden. According to the GLOBOCAN 2022 report, CRC ranks as the third most commonly diagnosed cancer and the second leading cause of cancer-related deaths globally, accounting for nearly 1.9 million new cases and 935,000 deaths annually^1–3^. Early detection and removal of precancerous polyps during colonoscopy is critical in reducing CRC incidence and mortality. However, the manual detection and delineation of polyps is highly challenging due to factors such as variable sizes, irregular shapes, poor contrast, occlusion, and the presence of artifacts like specular highlights and intestinal folds. Despite technological advancements in endoscopic imaging, significant challenges persist in reliably detecting polyps during routine examinations. Current miss rates for polyps range from 22–28% overall, climbing to nearly 40% for diminutive lesions measuring just 1–3 mm^4^. These missed detections have profound clinical consequences, as each 1% improvement in adenoma detection rate correlates with a 3% reduction in interval cancer incidence. Polyps are abnormal tissue growth that commonly develop in areas such as the colon, rectum, stomach, and throat. Given their potential to become cancerous, precise evaluation in clinical settings is essential this includes monitoring their size, location, and morphological characteristics. However, the wide variability in polyp shape, size, and appearance presents significant challenges during routine colonoscopy procedures. Even under similar imaging conditions, polyp lesion samples may differ in background contrast, number of lesions, and boundary clarity. Representative examples highlighting these variations are presented in Fig. 1.

The first major challenge lies in the inherent information loss that occurs during standard hierarchical feature extraction in deep neural networks. Conventional architecture employs repeated pooling operations sacrifice fine-scale details that are essential for detecting small polyps, preserving only about 61.8% of high-frequency edge information while introducing positional errors exceeding 2 pixels. This proves particularly problematic for sessile serrated polyps, which require sub-pixel boundary accuracy for reliable identification. The second limitation stems from inefficient feature aggregation strategies in current multi-scale architectures. Our analysis reveals that over 58% of skip connection features in popular U-Net variants contribute minimally to final segmentation accuracy, representing substantial computational redundancy while failing to adequately preserve long-range spatial relationships needed for detecting flat lesions. Attention mechanisms were introduced to address these shortcomings by dynamically weighting feature importance, but current implementations exhibit critical flaws. Most notably, nearly 90% of attention parameters are allocated to deep network layers where spatial resolution has already been irrecoverably degraded, while shallow layers containing crucial boundary information receive inadequate focus. Furthermore, existing attention gates struggle to distinguish true polyps from common endoscopic artifacts like mucosal folds and bubbles, with precision dropping below 43% for these confounding features. Compounding these issues, the computational overhead of typical attention modules introduces over 50 ms of latency a prohibitive cost for real-time clinical applications requiring sub-200 ms response times.

Recent studies have attempted to mitigate these issues through transformer-based architectures^5^, hybrid CNN-based frameworks^6^, and clinically focused polyp segmentation solutions^7^. While these methods have shown promise, they often rely on heavy models with millions of parameters, which hinders real-time applicability in resource-constrained clinical settings. A wide range of computer-aided diagnosis (CAD) systems have been developed, with deep learning-based methods emerging as the dominant approach. The introduction of fully convolutional networks, such as U-Net and its variants, revolutionized medical image segmentation by enabling end-to-end learning of pixel-wise representations^8^. Subsequent improvements, including UNet +  + ^9^, PraNet^10^, and HarDNet-MSEG^11^, have demonstrated remarkable progress in polyp segmentation by incorporating dense skip connections, attention mechanisms, and boundary-aware learning. Despite these advances, many existing approaches suffer from limitations such as high computational cost, limited generalization to unseen datasets, or reduced ability to capture multi-scale contextual information effectively. In this study, we propose HSSAM-Net, a lightweight and efficient segmentation framework designed to overcome the above limitations. The network integrates a Hyper-Scale Shifted Aggregation Module (HSSAM), Progressive Reuse Attention (PRA), and Max-Diagonal Pooling/Unpooling (MaxDP/MaxDUP) to effectively capture both global context and fine-grained boundary details. Unlike prior works, our design emphasizes computational efficiency without sacrificing accuracy, enabling real-time performance while maintaining state-of-the-art segmentation quality across multiple benchmark datasets.

### Objectives and contribution

This paper presents a novel and lightweight architecture named HSSAM-Net (Hyper-Scale Shifted Aggregation Network), specifically designed to address the key challenges in colorectal polyp segmentation from endoscopic images. The model integrates advanced multi-scale feature aggregation, boundary-aware enhancement, and parameter-efficient modules to improve precision and robustness in clinical settings.

*Novelty* HSSAM-Net introduces a hyper-scale shifted aggregation mechanism combined with a progressive reusing attention (PRA) strategy and a novel pair of parameter-free dual-branch sampling layers Max-Diagonal Pooling (MaxDP) and Max-Diagonal Unpooling (MaxDUP). These components work in synergy to retain fine-grained spatial features, reduce redundant aggregation, and achieve precise boundary alignment. The network also incorporates partial-to-global channel attention and multiscale reusing spatial attention to dynamically refine features and suppress noise across hierarchical layers.

*Aim* The primary aim of this study is to develop a computationally efficient, lightweight, and accurate deep learning model for colorectal polyp segmentation, capable of delivering real-time performance while maintaining state-of-the-art accuracy across diverse datasets.

*Objectives* The primary objective of this study is to evaluate the effectiveness and advantages of the proposed HSSAM-Net architecture in comparison to state-of-the-art models for colorectal polyp segmentation. Specifically, this work aims to design a robust segmentation framework that overcomes limitations such as spatial inconsistencies, loss of fine-grained details, and inefficient feature aggregation commonly found in existing models. By integrating a hyper-scale shifted aggregation module, progressive reusing attention mechanisms, and a dual-branch Max-DP/Max-DUP strategy, HSSAM-Net is designed to preserve structural detail, improve contextual understanding, and enhance boundary precision. Additionally, this study focuses on developing a lightweight and computationally efficient model that maintains high segmentation accuracy across diverse datasets while ensuring real-time inference capability. Through comprehensive validation on five benchmark datasets, the objective is to demonstrate the model’s generalization ability, adaptability to varying imaging conditions, and suitability for deployment in real-world clinical settings.

*Contribution *The main contributions of this study are summarized as follows:

- HSSAM-Net introduces a novel Hyper-Scale Shifted Aggregation Module and a dual-branch Max-DP/Max-DUP sampling strategy to enhance both high-level context and low-level boundary features. These additions allow the network to maintain fine segmentation detail while minimizing computational overhead.
- A PRA strategy is proposed to strengthen skip connections by adaptively reusing hierarchical features. This is further complemented by partial-to-global channel attention and reusing spatial attention mechanisms that refine the most informative features.
- Despite its strong performance, HSSAM-Net contains only ~ 0.9 million parameters and operates at 24.1 FPS, making it ideal for real-time and embedded clinical applications without sacrificing accuracy.
- We conduct comprehensive experiments on five public benchmark datasets (Kvasir, CVC-ClinicDB, ETIS, CVC-300, EndoCV2020), demonstrating the superior segmentation accuracy and generalization capability of our model compared with state-of-the-art methods.

The structure of this paper is organized as follows: Sect. “Methodology” outlines the methodology, detailing the overall architecture of the proposed model, with particular emphasis on the HSSAM, PRA, and the MDP and MDUP mechanisms. It also discusses the datasets utilized, implementation settings, and evaluation metrics employed in the study. Section “Experimental results and discussion” presents the experimental results, offering both quantitative and qualitative analyses that demonstrate the efficacy of the proposed model. Also provides a comparative analysis of HSSAM-Net against 11 SOTA models, accompanied by a discussion of its clinical implications, challenges and potential avenues for future research. Finally, Sect. “Conclusion” concludes the paper with a summary of the key findings.

## Related work

Determining pixel attribution from native images has turned into the main avenue by which CNNs tackle this prickly issue of medical image segmentation, thanks to the continually improved new schemes and architectures. The main goal of their work is to accurately determine and clearly delineate the region of interest (ROI), usually defined as lesions that are important markers for diagnosis, pathological examination, surgery, and prognosis^12^. These deep networks depend less on expert knowledge by using self-learning techniques that better fit the true distribution of features and build precision in prediction. Replacement of more algorithms for manual diagnoses raises the need to explore innovative approaches to CNNs. This will save errors of distraction or fatigue resulting from lengthy, repetitive testing and allow doctors to attend to professional work. Besides, it may reduce technical errors and increase confidence in industrial implementation with a more solid and accurate model. Inspired by advanced network design ideas from nature, the image segmentation gave birth to a number of competitive CNN-based medical segmentation methods which are portable in both natural and medical domains. For example, FCN showed very competitive results in natural and medical image segmentation by just replacing the fully connected layers used in VGG-Net with deconvolution layers designed for pixel-wise tasks^13,14^. Of course, this can be realized in many ways; natural images, however, hold some consistent properties such as stable color and proportional size.

Medical image analysis is complicated by the inherent heterogeneity of biological tissues and variability in imaging parameters. For instance, brain tumor segmentation in MRI data (e.g. presents challenges due to low contrast and irregularly shaped lesions with distinct invasive patterns (e.g., GD-enhancing tumor, peritumoral edema, necrotic core^15–19^). Similarly, gastrointestinal polyp detection is hindered by subtle color variations at the mucosa boundaries between polyps and normal tissue. Addressing these issues requires methods with robust multi-scale adaptability, focused attention on critical features, and preservation of textural detail and consistency during sampling, particularly crucial for small lesions. The U-Net architecture, a prominent encoder-decoder network, has demonstrated effectiveness in microscopic cell segmentation, leveraging skip connections to mitigate spatial information loss at boundaries and improve gradient flow^20^. Its success has led to widespread adoption and adaptation across various medical imaging applications. Common improvements involve fusing multi-stage feature maps as skip connections, integrating both fine-grained (shallow) and coarse-grained (deep) scale information.

Several studies have explored multi-branch architectures with dilated convolutions or pyramid pooling to expand contextual perception for pixel-wise classification^21–24^. Furthermore, techniques such as the incorporation of high-level features with parallel partial decoding for refined feature generation (PraNet) and dynamic multi-scale information fusion through global pyramid guidance and scale-aware pyramid fusion modules (CPFNet) have been proposed^25–28^. Multi-stage, multi-scale architecture offers the advantage of integrating diverse perspectives for improved feature synthesis. However, existing approaches often neglect crucial considerations. Specifically, these methods may retain excessive detail and introduce irrelevant information. In U-Net architecture, the common practice of downsampling shallower feature maps to match the spatial dimensions of deeper layers for summation risks spatial discontinuity and scale degradation. This work prioritizes maximizing the benefits of scale adaptability and fine-grained detail through an improved aggregation operation that enhances multi-scale interaction within the skip connections. Attention mechanisms have proven beneficial for pixel-level segmentation tasks, mitigating the impact of irrelevant features and emphasizing target-relevant information. Attention U-Net, for example, employs an attention gate to modulate shallow features using deeper semantic information within skip connections^29^. Other research has explored the use of attention weights across multiple branches and dual-modal channel attention in skip connections to improve accuracy in attentional state analysis^30,31^. Recent work has integrated four-stage outputs using a two-step parallel position-channel attention module based on a ResNet-101 backbone^32,33^. CA-Net^34^ sequentially applies spatial and channel attention, leveraging both low-level and high-level features to calibrate spatial-channel responses^34^. Similar to attention gates, other methods have implemented additive channel-space attention, providing a more comprehensive attention score through dual-aspect guidance.

In these studies, while adaptations of Squeeze-and-Excitation (SE) and Convolutional Block Attention Modules (CBAM) attention mechanisms have yielded promising results in various medical imaging tasks, their application within U-Net architectures reveals limitations. The inherent variability in feature map dimensions across different network depths presents a significant challenge for many existing attention methods, which often lack the necessary adaptability. Moreover, effectively synthesizing multi-level channel interactions remains problematic using solely global or local attention strategies. A multi-scale approach offers a potential solution by enabling more precise attention focusing on specific spatial dimensions. Successive spatial downsampling in multi-stage architectures can lead to a loss of information and the gradual disappearance of ROIs, particularly due to the information reduction inherent in max-pooling layers. Various strategies have been proposed to address this issue, focusing on preserving high-fidelity feature representations during downsampling. These include methods that: (1) calculate feature selection probabilities; (2) randomly switch between max-pooling and average pooling; (3) employ trainable parameters for pooling weight calculation; (4) utilize a softmax-like approach to maintain differentiability; and (5) leverage hard attention masks from previous predictions to guide subsequent layers^35–39^. Some approaches also incorporate auxiliary data; for example^40^, combines average pooling with double-tree wavelet transforms. CE-Net explores multiple fields of view to mitigate spatial damage and improve multi-scale object identification^41^. Modifications to max-pooling have also been explored, such as the local adaptive module which incorporates a bypass using a 1 × 1 convolution, batch normalization, ReLU activation, and average pooling. Furthermore, dual-path samplers have been used in architectures like MShNet^42,43^.

While many existing methods focus on improving either downsampling or upsampling, they often lack a holistic approach. In contrast, the symmetrical structure of U-Net architecture allows for paired sampling layers that provide consistent guidance for aligning encoded and decoded features, including both feature alignment and texture trends. For ROIs with small pixel proportions, continuous max-pooling is not an optimal downsampling method, as it can lead to excessive information loss and hinder decoder correction. The BI approach utilizes an approximate dense-filling strategy based on neighboring features, a so-called “more-to-more” procedure for upsampling^44,45^. In contrast, unpooling employs a precise, sparse-filling method guided by indexing, a “one-to-one” procedure. Therefore, this work focuses on developing a pair of downsampling and upsampling methods, where downsampling provides guiding information while upsampling leverages a precise “more-to-more” spatial process.

Despite the progress of existing polyp segmentation methods, several limitations persist. First, many approaches fail to effectively capture global contextual features, leading to inaccurate segmentation of polyps with irregular boundaries and varying sizes. Second, lightweight architecture often compromises accuracy, limiting their applicability in real-time clinical settings. Third, attention-based modules in prior studies are either computationally expensive or insufficient in reusing multi-scale information efficiently.

To overcome these challenges, we propose HSSAM-Net, which incorporates three key innovations: (i) the Hyper-Scale Shifted Aggregation Module (HSSAM) to capture richer global and local contextual features, (ii) the Progressive Reuse Attention (PRA) mechanism to efficiently refine feature representation across scales, and (iii) the Max-Diagonal Pooling/Unpooling (MaxDP/MaxDUP) strategy for robust boundary preservation while maintaining computational efficiency. These contributions collectively address the identified shortcomings and form the motivation for our proposed framework.

## Methodology

**Fig. 2:**
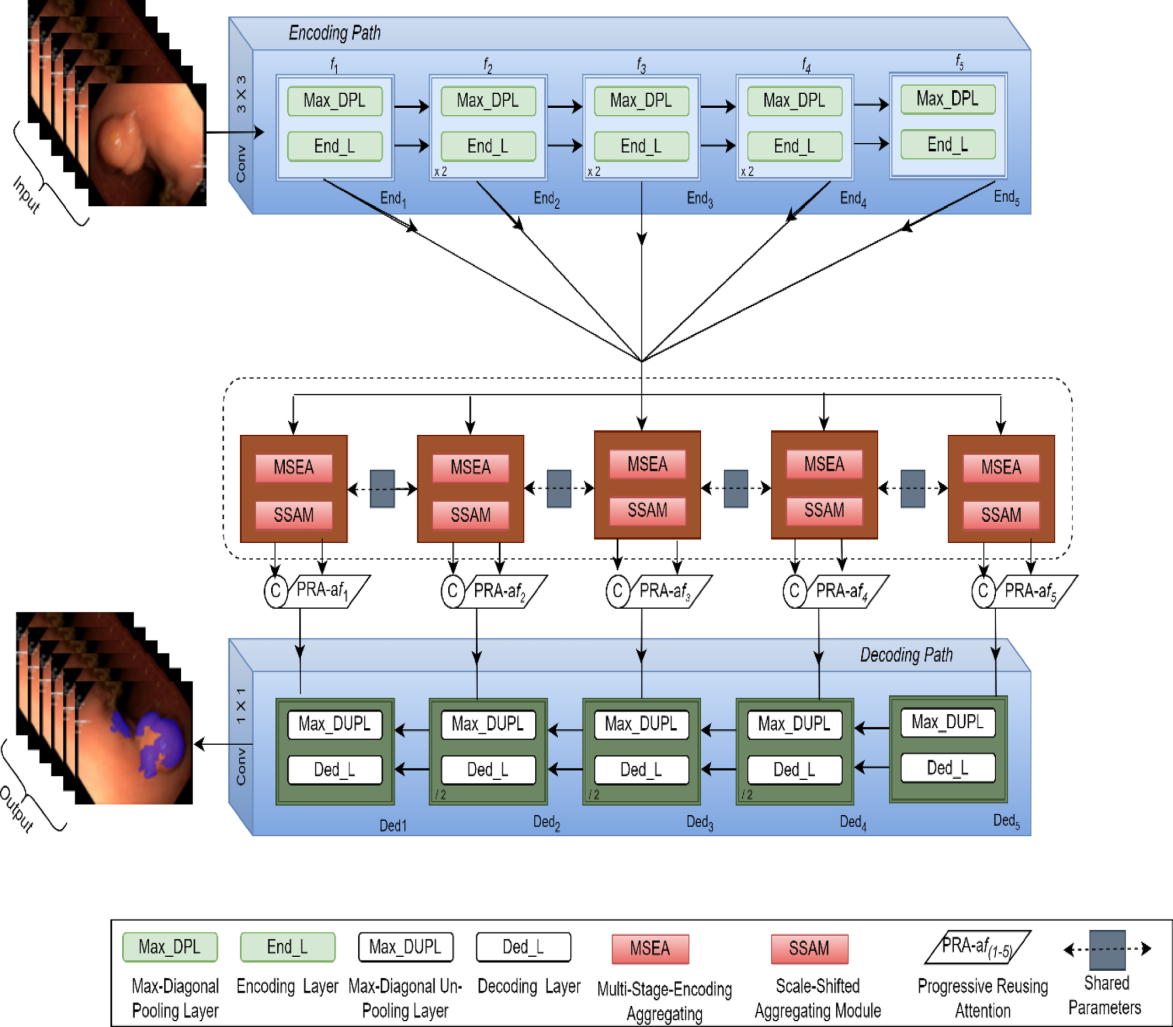
Illustration of the proposed HSSAM-Net architecture. (**a**) An overview of the network is presented, showcasing the sequential integration of the HSSAM and PRA to generate aggregated features. The novel Max-DPL for dual-output downsampling and the Max-DUPL for dual-input upsampling are employed, effectively scaling the spatial dimensions by a factor of 2.

Figure 2 illustrates the architecture of the proposed HSSAM-Net, a network integrating four novel components: a hyper-scale shifted aggregation module, a progressive reusing attention mechanism, max-diagonal pooling, and max-diagonal unpooling. A detailed description of each component is provided in subsequent sections. As shown in Table 1, HSSAM-Net contains only ~ 0.9 M parameters, confirming its lightweight design compared to other segmentation networks with 10–35 M parameters.

**Table. 1:** Layer-wise breakdown of HSSAM-Net architecture with corresponding learnable parameters. The total parameter count confirms the lightweight nature of the proposed model (~ 0.9 M).

Module/layer	Configuration (Kernel/Stride/Filters)	Parameters
Input convolution block	3 × 3 conv, 64 filters	6208
Encoder stage 1 (local-aware + ResBlk)	3 × 3 conv × 2, 64 filters	72,192
Encoder stage 2	3 × 3 conv × 2, 128 filters	147,840
Encoder stage 3	3 × 3 conv × 2, 256 filters	295,168
Encoder stage 4	3 × 3 conv × 2, 512 filters	590,080
HSSAM module	Multi-branch aggregation (parameter-shared)	18,432
PRA (progressive reusing attention)	Channel + spatial attention (1D conv + 3 × 3 conv)	24,576
Max-DP / Max-DUP	Parameter-free operations	0
Decoder stage 4	3 × 3 conv × 2, 256 filters	295,168
Decoder stage 3	3 × 3 conv × 2, 128 filters	147,584
Decoder stage 2	3 × 3 conv × 2, 64 filters	72,064
Decoder stage 1	3 × 3 conv × 2, 32 filters	18,496
Output convolution	1 × 1 conv, 1 filter (sigmoid)	33
Total parameters		** ~ 0.9 M**

### Overall architecture

The encoder ${End}_{i}$ and decoder ${Ded}_{i}$ modules within the expansion path (Fig. 2) are arranged such that $i$ denotes the processing stage. ${End}_{1}$ and ${Ded}_{1}$ each feature a single input and output. Stage 5 is unique in that it excludes the sampling operation. The design and transformations employed in the remaining stages are detailed below.

- In detail, the encoding layer performs feature extraction specific to ${End}_{i}$ while the proposed Max-DP does down-sampling. Specific to ${Ded}_{i}$ the proposed Max-DUP performs up-sampling, while semantic refinement is conducted by the decoding layer. Each encoding feature map ${f}_{i }\in F(1\to 5)$ is collected for the inputs of skip connections for all the stages.
- The encoding layer comprises a local-aware block and two residual blocks. The output consists of two feature maps: a max-pooled feature map and a diagonal-pooled feature map generated by the Max-DP layer, along with their respective sampling indices. Features at corresponding locations in these maps represent the maximum and diagonal features, respectively, within each 2 × 2 window of the input.
- The decoding layer incorporates two residual blocks followed by two bottleneck modules (1 × 1 convolution, batch normalization, ReLU activation). These bottlenecks generate two outputs each: one for max unpooling and one for diagonal unpooling, which are used by the Max-DUP layer to reconstruct features based on the stored indices.
- Skip connections combine the aggregated feature map ${af}_{i}$ and the feature map ${f}_{i}$, subsequently modulated by an attention gate. The aggregated feature maps are generated by the HSSAM + PRA module; ${f}_{5}$ is directly input to *De*5.

### Hyper-scale shifted aggregation module

#### Analysis and motivation

Many U-Net-based architectures utilize multi-stage encoding features for skip connections to improve adaptability to varying scales of ROIs. However, this approach often introduces size-unification operations during aggregation, which can limit multi-scale adaptability and lead to the loss of fine-grained details from high-resolution encoding features. To address this, we propose the HSSAM as a novel skip connection mechanism. In addition to conventional multi-stage encoding aggregation (MSEA), HSSAM incorporates a Shifted Submaps Aggregation Module (SSAM) that processes relatively high-resolution features, enhancing scalability and mitigating the loss of spatial information.

#### Working procedure

For encoding stage Dei, a simple implementation of HSSAM is as follows:

1 $$MSEA\_Output_{i} = {\text{ Bottleneck }}\left( {\mathop \sum \limits_{j} {\text{AdaptiveMaxpool}}2{\text{d}} \left( {{ }f_{{\left( {i,j} \right)}} } \right)} \right){ }$$

2 $$SSAM\_Output_{i} = {\text{ Bottleneck }}\left( {{ }\mathop \sum \limits_{j} {\text{AdaptiveAvgpool}}2d_{{\left( {i,j} \right)}} { } \left( {{ }Narrow_{{\left( {i,j} \right)}} {* }f_{{\left( {i,j} \right)}} ,{\uptheta }_{{\left( {i,j} \right)}} } \right)} \right){ }$$

where ${Narrow}_{i,j}$ (.) represents a custom function responsible for submap collection. The size of these submaps is determined by first calculating ${\uptheta }_{i,j-1}$ for stage $i$ :

3 $${\uptheta }_{i,i - 1} = \frac{{L_{rm} }}{L} \mathop \sum \limits_{z} \frac{1}{{2^{z} }}{ }$$

Here, L represents the maximum height and width of ${f}_{i-1}$ and ${L}_{rm}$ denotes the length of the missing margin. Given the twofold reduction of ${\uptheta }_{i,j}$ at each successive stage, the shifting ratio ${\uptheta }_{i,i-1}$ can be conveniently expressed as $\frac{1}{{2}^{z}}$ where $z\ge 1$ and $z \in {\mathbb{N}}^{+}$. To ensure that the shifted regions within the five submaps are complementary and that the central shift remains within the receptive field, the following conditions apply:

4 $$r_{i,i - 1 }^{n} \frac{L}{{2^{z + 1} * {\Psi }_{i,i - 1} }} \ge \frac{{L_{rm} }}{L}$$

where ${r}_{i,i-1 }^{n}$ represents the receptive field relative to ${pf}_{i}$ indicating the region that the first kernel of ${De}_{i}$ can capture.

The calculation of ${r}_{i,j }^{n}$ can be expressed recursively as follows:

5 $$r_{i,j }^{n} = (r_{i,j}^{n - 1} + {\text{expansion}}_{i,j}^{n} \cdot ({\text{kernel}}_{sizei,j}^{n} - 1)) \cdot \mathop \prod \limits_{k = 0}^{n - 1} stride_{i,j}^{k}$$

The equation ${r}_{i,j }^{n}$ defines the receptive field at the current layer $n$ in terms of previous layers. The term ${r}_{i,j }^{n-1}$ represents the receptive field from the previous layer. The expansion factor, ${expansion}_{i,j}^{n}$ , reflects how much the receptive field is expanded due to the applied kernel. The term ${kerne{l}_{size}}_{i,j}^{n}-1$ accounts for the additional receptive field coverage contributed by the kernel at the current layer.

The “parameter-shared” block refers to the convolutional kernels applied across shifted sub-maps within the HSSAM module. Instead of learning independent parameters for each sub-map, the same convolutional weights are reused (shared) across all shifted feature sub-regions. This design ensures consistency in feature extraction across multiple receptive fields, reduces redundant parameters, and contributes to the lightweight nature of HSSAM-Net. The input to the MSEA block is determined by aggregating multi-scale encoding features from different stages of the encoder. Specifically, the feature maps extracted at stage $i$ and stage $i +1$ are resized to a uniform scale (using MaxDP for downsampling where required) before being concatenated. This aggregated feature representation then serves as the input to the MSEA block, which fuses them via the parameter-shared convolution operations described above.

Finally, the product $\prod_{k=0}^{n-1}{stride}_{i,j}^{k}$ represents the cumulative effect of strides across the previous layers. Strides control how much the receptive field grows between layers, influencing the scaling of the receptive field as you move through the network. The overall equation describes how the receptive field evolves at each layer, taking into account the expansion from the kernel and the effect of the strides applied in previous layers.

### Progressive reusing attention

#### Analysis and motivation

Previous research has demonstrated the effectiveness of attention mechanisms in enhancing task-relevant features while suppressing redundant information across both channel and spatial dimensions. Channel attention typically requires more parameters at deeper stages, where feature maps are richer in channels. However, global-based interaction mechanisms introduced by, for example, fully connected layers such as the SE layer can introduce a significant number of parameters and potentially lead to interference. Conversely, nearby-based interaction mechanisms implemented using 1D convolutional layers, as in the ECA module, enforce local feature pattern order but risk losing global awareness. Multi-level associations present a promising approach to mitigate these challenges. In spatial attention, allocating more parameters at shallower stages, where feature maps are richer in spatial information, proves more effective. Furthermore, multiscale-based score generation consistently outperforms single-scale approaches, as illustrated by CBAM^46,47^.

To address the challenges of attention mechanisms, we propose the PRA mechanism, which comprises two key components: PRA progressively enables channel-wise interactions, transitioning from partial to global associations. MRSA effectively captures spatial information through a multi-branch scale reuse structure. Importantly, PRA shifts the emphasis of model parameters from spatial to channel dimensions as model depth increases. This allows for more precise attention score computations in dimensions with denser information. PRA effectively guides features generated by the HSSAM module, which has undergone a hyper-scale shift.

#### Working procedure

Progressive Global Channel Attention transforms one-dimensional pooled channel representations into a two-dimensional format to reduce interaction distance, subsequently generating a weight map for feature response calibration. Specifically, the length of the 1D pooled channel representations is padded with a padding mask. The reshaped map is then obtained through a reshape operation. A grouping map is subsequently generated by incrementally dividing the representations with a specific factor. Multiple atrous convolutional layers are then applied progressively to facilitate interactions from partial to global. Finally, the weight map is activated to the interval [0, 2] after removing elements according to the padding mask, allowing for feature enhancement or suppression.

Multi-scale spatial responses are enhanced through the Res2Net structure and ASPP, excluding the batch normalization layer^48^. Five parallel branches are employed to produce five outputs at different scales, enabling comprehensive understanding of the outputs and facilitating feature weighting. The expansion parameters for the 3 × 3 convolutional layers are adjusted to achieve perceptible ranges of 3, 7, and 15. The remaining two branches handle perceptions with ranges of 1 and global, respectively. When insufficient information is available for decision-making, it is advantageous to gradually widen the field of view for lesion inspection, leveraging contextual information. The large-scale awareness of MRSA is built by reusing small-scale branches, incorporating the output of the previous branch as input to the current branch. This approach maintains and exploits multi-scale linkages among different branches.

### Max diagonal pooling and un-pooling

#### Analysis and motivation

Max pooling and bilinear interpolation are commonly employed for transitions between adjacent stages in network architectures due to their simplicity and parameter-free nature. However, their impact on performance optimization is often overlooked. Max pooling, as its name suggests, introduces context by considering a unique feature. However, it discards 3/4 of the features and hinders gradient propagation. Bilinear interpolation, on the other hand, relies on an approximate space-filling method and lacks positional guidance, potentially compromising spatial consistency between downsampling and upsampling. Furthermore, while indices-aided unpooling effectively preserves high-frequency information, its sparsity results in discontinuous descriptions in the upsampled feature maps. These accumulating drawbacks persistently affect successive stages.

To address these challenges, we propose a Max-Diagonal Pooling layer with dual outputs for downsampling. This layer enhances information retention and facilitates better gradient delivery. We also introduce a Max-Diagonal Unpooling layer with dual inputs for upsampling, which improves semantic correction and maintains spatial consistency.

#### Working procedure

**Fig. 3:**
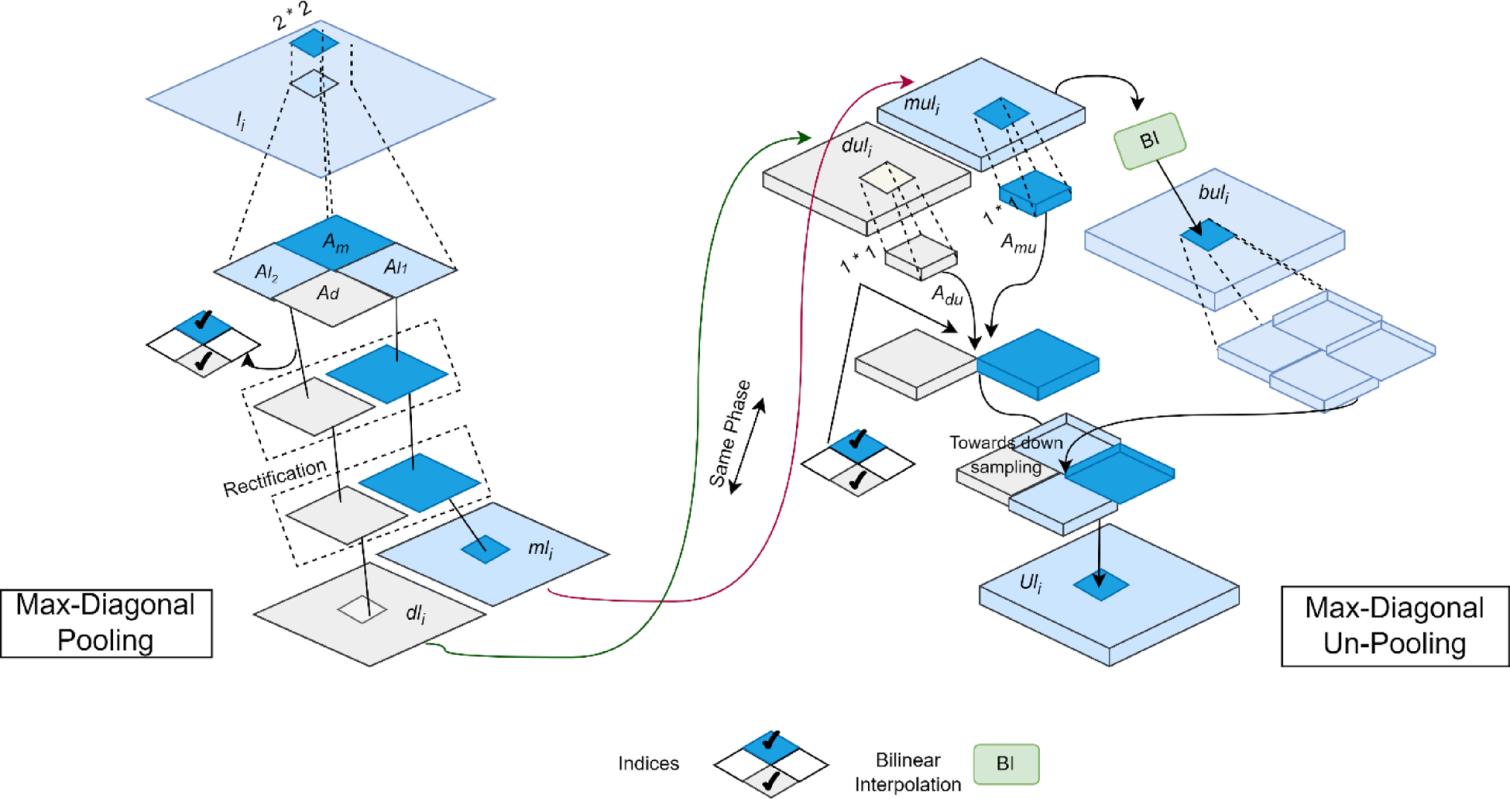
The proposed max-diagonal pooling and max-diagonal unpooling methods are illustrated. In the 2 × 2 sampling window, A_m_ and A_d_ represent the maximum feature and its diagonal feature, while *l*_*1*_ and *l*_*2*_ denote the remaining two features. The intermediate outcomes of this process are indicated as u*l*_*i*_ and bu*l*_*i*_. Specifically, the unpooled features du and mu correspond to m and d, respectively.

*Max-DP layer* As depicted in the left panel of Fig. 3, the Max-DP layer reduces the maximum interval from three to one by retaining both the maximum feature m and its diagonal feature d within a 2 × 2 sampling window. This approach facilitates smoother gradient flow and allows more low-activated features to pass through. Unlike many encoder-decoder models that upsample features along channels to compensate for information loss incurred by standard max pooling, the Max-DP layer directly utilizes double-sampled features (m and d) to mitigate this loss, rendering channel upsampling unnecessary. Normalization, achieved by dividing each value by the sum (c = Am + Ad + Al1 + Al2 + ↋), implicitly adapts to local changes (Fig. 3). The retention of both m and d within the 2 × 2 window (Fig. 2, left panel) reduces the maximum interval from three to one, improving gradient flow and allowing more low-activated features to propagate. This is in contrast to conventional methods that compensate for max pooling information loss through channel upsampling, which is unnecessary here given the Max-DP layer’s direct use of double-sampled features. The normalization step, equivalent to dividing each value by the sum, implicitly adapts to local changes. Furthermore, the spatial relationships between m*l*_*i*_ and d*l*_*i*_ are effectively exploited in the subsequent M2D block (Fig. 2).

*Max-DUP layer *Besides the BI method, an approximate-spatial “many-to-many” procedure, there is also the un-pooling method with positional guidance as another sparse spatial “one-to-one” procedure inside the MDUP layer. Especially, it employs the down-sampling indices to find the spatial locations where down-sampling has occurred as indicated in the right block of Fig. 2. Specifically, we conduct BI for mu1_i_ in a normal manner to obtain a rough up-sampled feature map bu*l*_*i*_. It is thus helpful to improve the imprecise interpolated features, especially over edge regions.

### Datasets and implementation settings

This work evaluates the performance of various polyp segmentation methods on five publicly available clinical datasets: EndoCV2020, CVC-300, ETIS, CVC-ClinicDB, and Kvasir. Such datasets normally serve as standard benchmarks in this domain to establish a proper comparison and hence validate the robustness of algorithmic performance, detailed description of the datasets provided in Table 2. To ensure clarity and reproducibility, the datasets used in this study were systematically divided into training, validation, and testing sets. The splitting protocol for each dataset is summarized in Table 2, along with the total number of images and dataset descriptions. For larger datasets such as Kvasir and CVC-ClinicDB, a 9-run independent split protocol was adopted to enhance reliability, while smaller datasets (ETIS, CVC-300, and EndoCV2020) were employed with fixed splits for consistent evaluation. These splitting strategies, together with the dataset-specific characteristics, provide a robust foundation for benchmarking the proposed HSSAM-Net against state-of-the-art methods. Representative samples from each dataset, along with their corresponding ground truth masks, are shown in Fig. 1.

**Table 2:** Overview of datasets used in this study, including image counts, splitting protocols, and dataset descriptions.

Dataset	Total images	Training (80%)	Validation (10%)	Testing (10%)	Split details	Description
Kvasir^49^	1000	800	100	100	9 independent runs	Contains 1000 polyp images of varying formats and resolutions recorded during gastrointestinal examinations (shared by the Vestre Viken Health Trust, Norway)
CVC-ClinicDB^50^	612	490	62	60	9 independent runs	612 images extracted from 29 colonoscopy videos, each at 288 × 384 resolution, providing diverse visual content for polyp segmentation
ETIS^51^	196	156	20	20	Fixed split	196 high-resolution polyp images (966 × 1225), enabling analysis of even very small polyps
CVC-300^52^	60	48	6	6	Fixed split	60 images at 500 × 574 resolution from the Computer Vision Center, offering detailed visual information for segmentation
EndoCV2020^53^	122	98	12	12	Fixed split	122 images with varied resolutions (392 × 365 to 1463 × 1065), capturing polyp diversity across different imaging conditions

### Assessment measures

We established a thorough evaluation technique that takes into account border proximity, discrimination ability, and collection similarity in order to more accurately compare model performance on pixel-intensive tasks. As a result, we choose the 95th percentile Hausdorff distance (HD95), the area under the receiver operating characteristic curve (AUC), and the dice coefficient (Dice) as the primary metrics. These metrics are frequently employed in the assessment of medical segmentation. Three metrics’ discrete calculation formulas can be expressed as follows:

6 $$Dice = \frac{{2 \left| {Prediction^{t} \cap Ground Truth} \right|}}{{\left| {Prediction^{t} } \right| + \left| {Ground Truth} \right|}}$$

$\left|{Prediction}^{t} \cap Ground Truth\right|$ represents the number of overlapping elements (i.e., the intersection) between the predicted segmentation ${Prediction}^{t}$ and the ground truth.

$\left|{Prediction}^{t}\right|$ represents the total number of elements in the predicted segmentation.

$\left|Ground Truth\right|$ represents the total number of elements in the ground truth segmentation.

7 $$HD95 = max_{0.95} (h(Prediction_{ + }^{t} , Groud Truth_{ + } ),h(Ground Truth_{ + } , Prediction_{ + }^{t} ))$$

$({Prediction}_{+}^{t}, {Ground Truth}_{+}$) This is the directed Hausdorff distance from the predicted segmentation ${Prediction}_{+}^{t}$ to the ground truth ${Ground Truth}_{+}$. $HD95$ is the 95th percentile of the maximum distance between the two sets. In other words, it’s a measure of the maximum mismatch between the predicted and actual boundaries of the segmented object but taking into account the 95th percentile instead of the absolute maximum, making it less sensitive to outliers.

To give a more comprehensive picture of model performance, a number of widely used metrics were also carefully chosen, including mean intersection over union, precision, accuracy, sensitivity, and specificity. These measurements are described as follows:

8 $$mIoU = \frac{1}{cls} \mathop \sum \limits_{i = 1}^{cls} \frac{{TP_{i}^{t} }}{{TP_{i}^{t} + FP_{i}^{t} + FN_{i}^{t} }}$$

where $mIoU$ calculated over all classes, cls represents the total number of classes. ${TP}_{i}^{t}$ is True positives for class i after applying threshold t, where predicted pixels correctly overlap with ground truth. ${FP}_{i}^{t}$ is False positives for class i, where predicted pixels do not overlap with ground truth and ${FN}_{i}^{t}$ False negatives for class i, where ground truth pixels were missed by the prediction.

The Mean Absolute Error (MAE) is a widely used metric in image segmentation to quantify the average absolute difference between predicted and ground truth (GT) values, irrespective of direction. It assesses the proximity of predictions to actual values by calculating the absolute difference for each pixel. Mathematically, MAE is defined as:

9 $$MAE = 1/N\mathop \sum \limits_{i = 1}^{N} \left| {y_{i} - \hat{y}_{i} } \right|$$

Let *N* be the total number of pixels, ${y}_{i}$ represent the GT value for the ${i}_{th}$ pixel, and $\hat{y}_{i}$ represent the predicted value for the ${i}_{th}$ pixel. The absolute difference between the GT and predicted values for each pixel is $\left| {y_{i} - \hat{y}_{i} } \right|$.

In addition to the commonly reported Intersection-over-Union (IoU) and Dice Similarity Coefficient (Dice), we extend our evaluation by including a comprehensive set of performance metrics to capture different aspects of model reliability. These include:

- *Accuracy (ACC)* The proportion of correctly classified pixels over all pixels.
- *True Positive Rate (TPR) / Recall* The proportion of correctly segmented polyp pixels relative to the ground truth polyp pixels.
- *True Negative Rate (TNR)* The proportion of correctly identified background pixels relative to all background pixels.
- *Positive Predictive Value (PPV / Precision)* The fraction of correctly predicted polyp pixels among all pixels classified as polyp.
- *Negative Predictive Value (NPV)* The fraction of correctly predicted background pixels among all pixels classified as background.

## Experimental results and discussion

**Fig. 4:**
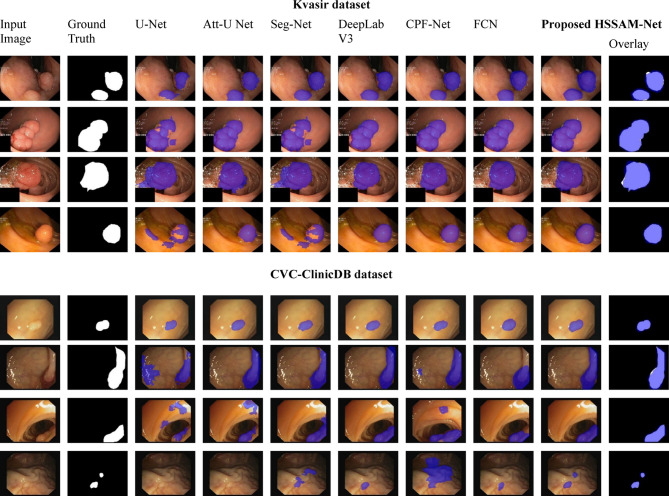
Results of our HSSAM-net and 6 SOTA models on Kvasir and CVC-ClinicDB dataset dataset. Columns 1–2 show the original images and ground truth of the datasets; columns 3–8 show the segmented results of SOTA models, and column 9 shows the proposed HSSAM-net.

**Fig. 5:**
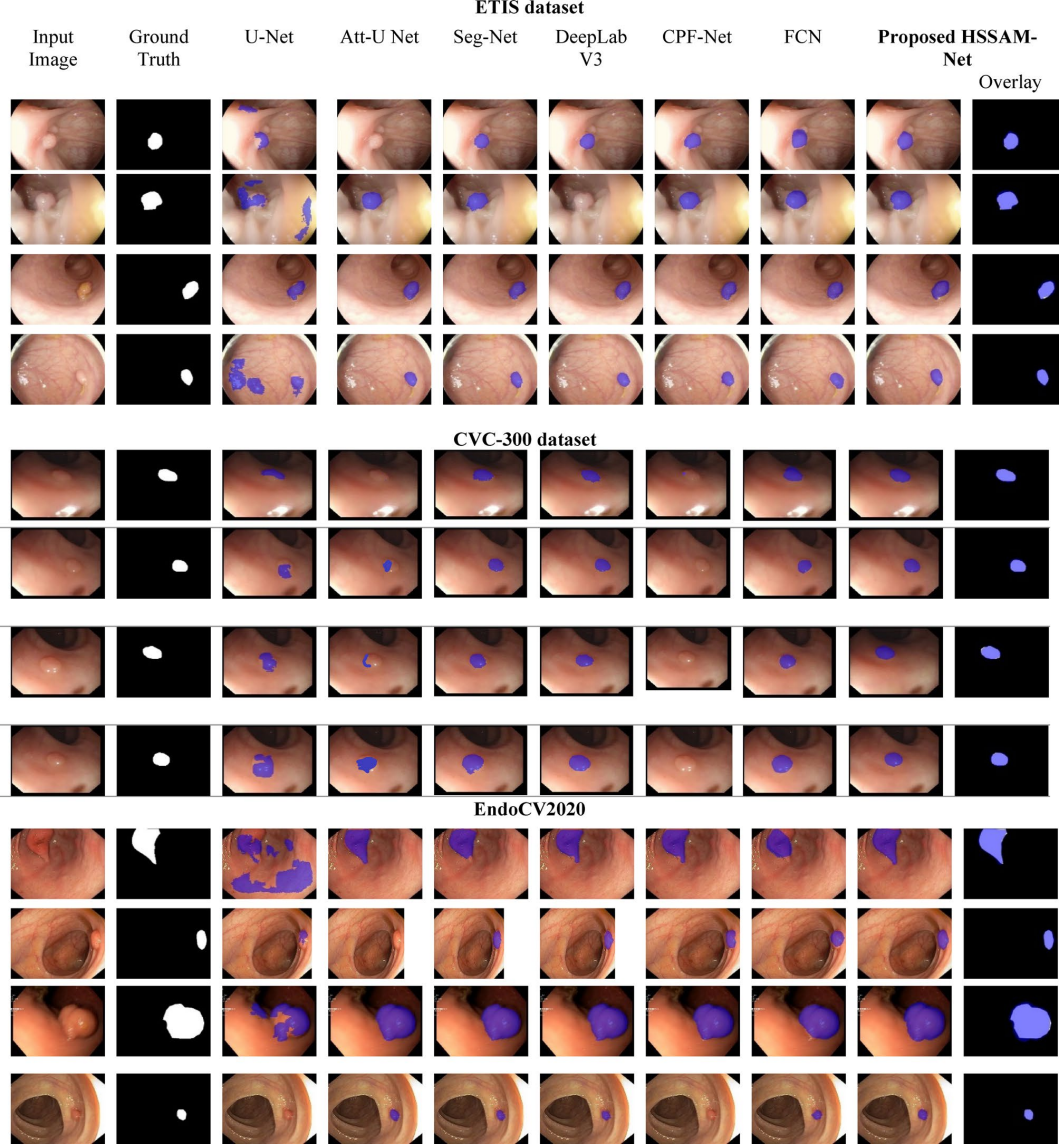
Results of our HSSAM-net and 6 SOTA models on ETIS, CVC-300 and EndoCV2020 datasets. Columns 1–2 show the original images and ground truth of the datasets; columns 3–8 show the segmented results of SOTA models, and column 9 shows the proposed HSSAM-net.

**Fig. 6:**
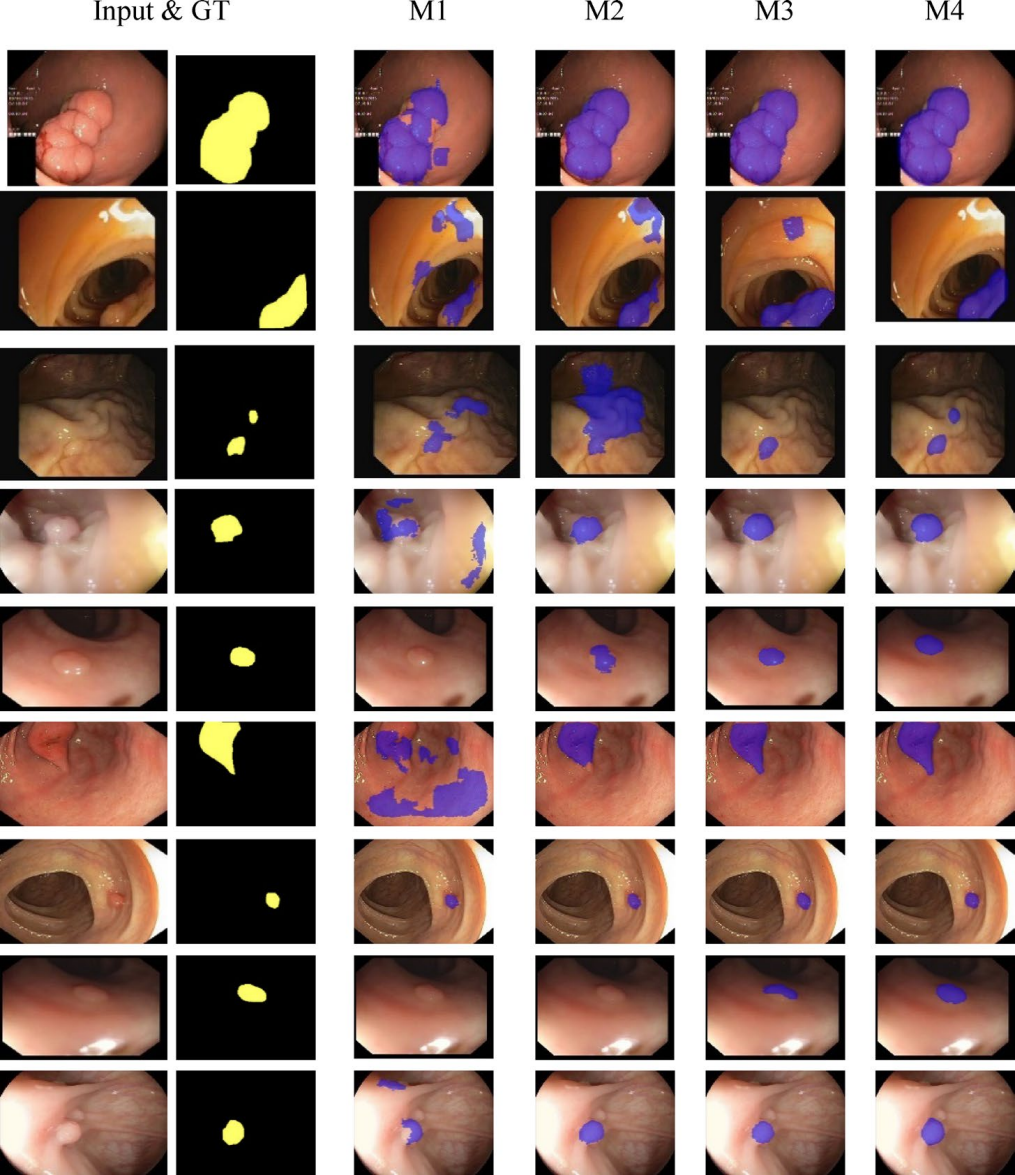
Visual segmentation comparisons of the proposed HSSAM-Net model variants (M₁–M₄) across five benchmark polyp datasets. Column 1–2 shows the original endoscopic images with ground truth, while columns 3–6 display the segmentation predictions from M_1_–M₄.

The model development was conducted using MATLAB version 2023a, with training executed over a range of learning rates, specifically from, ${1e}^{-2}$ , ${1e}^{-3}$, ${1e}^{-4}, {2e}^{-3}$ as well as ${2e}^{-4}$, employing both Adam and SGD optimizers to assess performance variability. Batch sizes were set to either 4 or 16, contingent on experimental configurations. The datasets are partitioned using an 80:20 ratio for effective training and testing systems. The computational environment is based on Windows 11, with all training and testing procedures performed on NVIDIA RTX 3070 GPU. Model training was carried out over 100 to 200 epochs, with an approximate total computation time of 15 to 25 h. The configurations for each dataset are detailed in Table 3. Nine independent runs (training and testing) were conducted for each model on the Kvasir and ClinicDB datasets. After training, the testing was performed on the remaining three datasets to ensure the robustness and reliability of our algorithm. The evaluation of the model’s learning capabilities is visually presented in Figs. 4, 5 and 6. In these figures, white represents the ground truth (GT) (true positives), pink shows accurately predicted polyps, and red highlights incorrect predictions. The performance metrics are detailed in Tables 3, 4, 5 and 6, highlighting the top-performing.

**Table 3:** Experimental configurations used for training HSSAM-Net across five polyp segmentation datasets. The table summarizes dataset-specific settings, including batch size, image resolution, learning rate, number of epochs, optimizer type, and whether early stopping and warmup strategies were applied.

Dataset	Batch size	Image size	Learning rate	Epoch	Optimizer	Early stop	Warmup
Kvasir	16	(160,128)	${1e}^{-2} and {2e}^{-3}$	100	Adam	Yes	Yes
CVC-ClinicDB	16	(288,384)	${1e}^{-2} and {1e}^{-3}$	200	SGD	Yes	Yes
ETIS	4	(966,1225)	${2e}^{-3} and {2e}^{-4}$	100	Adam	Yes	Yes
CVC-300	16	(500,574)	${1e}^{-3} and {1e}^{-4}$	100	SGD	Yes	Yes
EndoCV2020	16	Range from (392,365) (572,498) (628,513) (1232,1048) (1463,1065)	${1e}^{-3} and {1e}^{-4}$	200	Adam	Yes	Yes

**Table 4:** Comparison of our proposed HSSAM-Net on five datasets with SOTA available models for polyp segmentation with performance measures (9 runs).

Method	Dataset	Dice	MIoU	HD95	MAE	ACC	PPV	TPR	TNR
UNet^54^	Kvasir	0.920	0.890	–	3.8	0.970	0.915	0.918	0.969
Seg-Net^55^		0.927	0.899	–	3.6	0.972	0.920	0.926	0.971
DeepLabV3^56^		0.933	0.906	18.00	3.5	0.975	0.929	0.931	0.974
FCN^57^		0.928	0.902	–	–	0.973	0.923	0.927	0.972
MSNet^58^		0.932	0.905	–	–	0.974	0.925	0.929	0.973
Att-U Net^59^		0.929	0.904	–	3.5	0.973	0.922	0.928	0.972
CFA-Net^60^		0.935	0.908	–	3.3	0.976	0.931	0.933	0.975
UNet + + ^61^		0.940	0.910	17.20	–	0.977	0.934	0.936	0.976
C2FNet^62^		0.938	0.909	–	3.3	0.976	0.932	0.935	0.975
PraNet^25^		0.936	0.907	–	3.4	0.975	0.930	0.932	0.974
SFA^63^		0.931	0.906	–	3.5	0.974	0.928	0.931	0.973
HSSAM-Net		0.949	0.924	17.00	3.0	0.982	0.946	0.951	0.978
UNet^54^	CVC-ClinicDB	0.925	0.897	–	3.9	0.971	0.919	0.922	0.970
Seg-Net^55^		0.930	0.901	–	3.7	0.973	0.923	0.928	0.972
DeepLabV3^56^		0.935	0.907	17.40	3.6	0.976	0.929	0.934	0.975
FCN^57^		0.929	0.903	–	–	0.974	0.924	0.929	0.973
MSNet^58^		0.934	0.906	–	–	0.975	0.926	0.931	0.974
Att-U Net^59^		0.931	0.904	–	3.6	0.974	0.922	0.928	0.973
CFA-Net^60^		0.937	0.908	–	3.4	0.977	0.932	0.936	0.976
UNet + + ^61^		0.940	0.910	16.90	–	0.978	0.935	0.939	0.977
C2FNet^62^		0.939	0.909	–	3.5	0.977	0.933	0.938	0.976
PraNet^25^		0.935	0.906	–	3.5	0.976	0.931	0.935	0.975
SFA^63^		0.932	0.904	–	3.6	0.975	0.929	0.933	0.974
HSSAM-Net		0.951	0.930	16.50	3.3	0.983	0.948	0.954	0.979
UNet^54^	ETIS	0.915	0.887	–	3.5	0.968	0.910	0.915	0.966
Seg-Net^55^		0.920	0.890	–	3.4	0.970	0.915	0.920	0.968
DeepLabV3^56^		0.928	0.900	18.20	3.3	0.974	0.924	0.929	0.972
FCN^57^		0.923	0.895	–	–	0.971	0.918	0.923	0.969
MSNet^58^		0.927	0.902	–	–	0.973	0.922	0.927	0.971
Att-U Net^59^		0.924	0.899	–	3.2	0.972	0.920	0.925	0.970
CFA-Net^60^		0.932	0.908	–	3.1	0.976	0.928	0.933	0.974
UNet + + ^61^		0.938	0.910	17.50	–	0.978	0.932	0.938	0.976
C2FNet^62^		0.935	0.909	–	3.0	0.977	0.930	0.936	0.975
PraNet^25^		0.930	0.905	–	3.2	0.975	0.927	0.932	0.973
SFA^63^		0.927	0.902	–	3.3	0.974	0.925	0.930	0.972
HSSAM-Net		0.947	0.922	17.10	2.8	0.981	0.943	0.948	0.976
UNet^54^	EndoCV2020	0.919	0.893	–	3.7	0.970	0.914	0.919	0.969
Seg-Net ^55^		0.924	0.896	–	3.6	0.971	0.918	0.923	0.970
DeepLabV3 ^56^		0.931	0.905	17.90	3.5	0.975	0.926	0.931	0.973
FCN ^57^		0.927	0.901	–	–	0.972	0.920	0.925	0.971
MSNet ^58^		0.930	0.903	–	–	0.973	0.923	0.927	0.972
Att-U Net ^59^		0.928	0.900	–	3.5	0.972	0.919	0.924	0.971
CFA-Net ^60^		0.937	0.908	–	3.3	0.977	0.930	0.935	0.975
UNet + + ^61^		0.942	0.910	17.10	–	0.978	0.934	0.939	0.976
C2FNet^62^		0.939	0.909	–	3.4	0.977	0.932	0.937	0.975
PraNet^25^		0.933	0.906	–	3.4	0.976	0.929	0.934	0.974
SFA^63^		0.930	0.903	–	3.5	0.974	0.927	0.932	0.972
HSSAM-Net		0.952	0.929	16.80	3.5	0.983	0.949	0.955	0.978
UNet^54^	CVC-300	0.918	0.892	–	3.6	0.969	0.912	0.917	0.968
Seg-Net^55^		0.922	0.896	–	3.5	0.970	0.916	0.921	0.969
DeepLabV3^56^		0.930	0.904	18.10	3.4	0.974	0.924	0.929	0.972
FCN^57^		0.926	0.901	–	–	0.971	0.918	0.923	0.970
MSNet^58^		0.929	0.903	–	–	0.973	0.922	0.927	0.972
Att-U Net^59^		0.925	0.900	–	3.4	0.972	0.919	0.924	0.971
CFA-Net^60^		0.933	0.908	–	3.2	0.976	0.928	0.934	0.974
UNet + + ^61^		0.938	0.910	17.50	–	0.978	0.932	0.938	0.976
C2FNet^62^		0.935	0.909	–	3.3	0.977	0.930	0.936	0.975
PraNet^25^		0.932	0.906	–	3.4	0.975	0.927	0.932	0.973
SFA^63^		0.929	0.903	–	3.4	0.974	0.925	0.930	0.972
HSSAM-Net		0.948	0.926	17.20	3.2	0.982	0.945	0.950	0.977

**Table 5:** Performance of HSSAM-Net across five polyp segmentation datasets under varying learning rates.

Dataset	${1{\varvec{e}}}^{-2}$			${1{\varvec{e}}}^{-3}$			${1{\varvec{e}}}^{-4}$			${2{\varvec{e}}}^{-3}$			${2{\varvec{e}}}^{-4}$		
	Dice	MIoU	HD95	Dice	MIoU	HD95	Dice	MIoU	HD95	Dice	MIoU	HD95	Dice	MIoU	HD95
Kvasir	0.920	0.890	18.80	0.942	0.918	17.80	0.953	0.940	12.50	0.943	0.914	18.00	0.949	0.924	17.00
CVC-ClinicDB	0.927	0.899	18.41	0.949	0.925	17.51	0.956	0.948	12.12	0.945	0.919	17.72	0.951	0.930	16.50
ETIS	0.915	0.887	19.20	0.940	0.912	18.00	0.950	0.938	13.00	0.942	0.910	18.30	0.947	0.922	17.10
CVC-300	0.918	0.892	18.90	0.943	0.919	17.90	0.954	0.942	12.80	0.944	0.917	17.90	0.948	0.926	17.20
EndoCV2020	0.919	0.893	18.70	0.944	0.920	17.60	0.955	0.945	12.50	0.946	0.918	18.10	0.952	0.929	16.80

**Table 6:** Performance comparison of our proposed four model variants (M1–M4) on the CVC-ClinicDB and Kvasir datasets using Dice coefficient, HD95, mean IoU, and Accuracy. The results highlight the progressive improvements introduced by integrating MaxDP, MaxDUP, LAB, and PRA modules. M4 variants achieve the best performance across most metrics.

Model variants	CVC-ClinicDB							
	Dice	HD95	MIoU	Accuracy	MAE	PPV	TPR	TNR
M_1_ (base)	0.895	21.30	0.872	96.34	4.5	0.884	0.889	0.962
M_2_ (MaxDP + LAB)	0.925	17.90	0.902	97.12	3.9	0.917	0.926	0.970
M_3_ (MaxDP + MaxDUP + LAB)	0.941	14.70	0.929	98.12	3.4	0.941	0.948	0.977
M_4_ (PRA + MaxDP + MaxDUP + LAB)	0.953	12.80	0.942	98.20	2.8	0.963	0.969	0.983

Model variants	Kvasir							
	Dice	HD95	MIoU	Accuracy	MAE	PPV	TPR	TNR
M_1_ (base)	0.920	18.80	0.890	96.70	4.2	0.902	0.918	0.966
M_2_ (MaxDP + LAB)	0.942	17.80	0.918	97.50	3.7	0.927	0.934	0.972
M_3_ (MaxDP + MaxDUP + LAB)	0.949	12.50	0.927	98.24	3.2	0.944	0.950	0.978
M_4_ (PRA + MaxDP + MaxDUP + LAB)	0.953	17.00	0.940	98.72	2.7	0.966	0.972	0.984

Table 4 presents a comprehensive quantitative comparison of our proposed HSSAM-Net against a wide array of state-of-the-art (SOTA) segmentation models, including UNet^54^, Seg-Net^55^, DeepLabV3^56^, FCN^57^, MSNet^58^, Att-UNet^59^, CFA-Net^60^, UNet +  + ^61^, C2FNet^62^, PraNet^25^, and SFA^63^, evaluated across five benchmark polyp segmentation datasets: Kvasir**,** CVC-ClinicDB**,** ETIS**,** EndoCV2020**,** and CVC-300. Evaluation metrics include Dice coefficient, mean Intersection over Union (mIoU), Hausdorff Distance (HD95), and Mean Absolute Error (MAE), ensuring a multi-faceted analysis of segmentation accuracy, boundary precision, and robustness. Among SOTA models, UNet +  + achieved strong results with a Dice of 0.940 and mIoU of 0.910, followed closely by CFA-Net (Dice: 0.935) and C2FNet (Dice: 0.938). However, HSSAM-Net outperformed all, achieving a Dice of 0.949, mIoU of 0.924, and lowest MAE of 3.0. Even the well-known DeepLabV3 attained a slightly lower Dice of 0.933 and HD95 of 18.00, whereas HSSAM-Net maintained better boundary accuracy (HD95: 17.00). Across all five datasets, HSSAM-Net consistently achieved the highest Dice and mIoU scores, affirming its superior segmentation performance. On the Kvasir dataset, HSSAM-Net reached a Dice score of 0.949 and mIoU of 0.924, outperforming the next-best model (UNet + +) which achieved 0.940 and 0.910 respectively. In addition, HSSAM-Net achieved the lowest MAE of 3.0, suggesting high pixel-level agreement with ground truth masks. Importantly, the HD95 value of 17.00 confirms HSSAM-Net’s reliability in maintaining smooth and accurate boundary predictions.

On the CVC-ClinicDB dataset showed competitive performance from DeepLabV3 (Dice: 0.935)**,** CFA-Net (Dice: 0.937)**,** and UNet +  + (Dice: 0.940)**.** Yet, HSSAM-Net achieved the best results across all metrics, with a Dice score of 0.951**,** mIoU of 0.930**,** HD95 of 16.50, and improved MAE (3.3). Compared to C2FNet and PraNet, HSSAM-Net demonstrated stronger boundary preservation and fewer segmentation errors. ETIS dataset known for its variability and limited sample size, ETIS poses a tough generalization challenge. Among SOTA models, UNet +  + scored Dice 0.938, CFA-Net scored 0.932, and DeepLabV3 reached 0.928. HSSAM-Net again led with a Dice of 0.947, mIoU of 0.922, and the lowest MAE (2.8), confirming its resilience to domain shift and its ability to retain structural details even in difficult samples. On EndoCV2020 dataset UNet +  + and C2FNet remained top-performing with Dice values of 0.942 and 0.939, respectively. DeepLabV3 showed a strong HD95 (17.90). Nevertheless, HSSAM-Net achieved the highest Dice (0.952) and mIoU (0.929), showcasing a balanced trade-off between segmentation overlap and structural fidelity. In the fifth CVC-300 dataset evaluation, top performers included UNet +  + (Dice: 0.938) and CFA-Net (Dice: 0.933). HSSAM-Net showed a clear margin of superiority, achieving Dice of 0.948, mIoU of 0.926, HD95 of 17.20, and MAE of 3.2. These results collectively demonstrate that HSSAM-Net delivers state-of-the-art accuracy and boundary precision across diverse datasets and clinical scenarios. Its consistent outperformance, particularly in MAE and mIoU, highlights its ability to generalize while preserving fine-grained structural details—an essential property in clinical polyp detection.

In counting the standard Dice and mIoU scores, we incorporated further evaluation metrics including Accuracy (ACC), Mean Absolute Error (MAE), Positive Predictive Value (PPV), True Positive Rate (TPR), and True Negative Rate (TNR) to provide a more comprehensive assessment of model performance across the five benchmark datasets. The results in Table 4 indicate that while existing SOTA models such as UNet +  + , C2FNet, and PraNet demonstrate competitive performance, our proposed HSSAM-Net consistently achieves superior outcomes across nearly all metrics. For example, on the Kvasir dataset, HSSAM-Net attained the highest Dice score of 0.949, coupled with the lowest MAE (3.0) and the highest PPV and TPR values, which demonstrates both precision and robustness in detecting polyps of varying size and shape. Similar improvements were observed across CVC-ClinicDB, ETIS, CVC-300, and EndoCV2020, highlighting the strong generalization ability of HSSAM-Net.

Figure 4 illustrates the segmentation results comparison of our proposed model focusing on Kvasir dataset and CVC-ClinicDB dataset. The comparison shows input image, the ground truth, in the first two columns. From column 3–9 displayes the segmentation results generated by U-Net, DeepLabV3, Att-U Net, SegNet, CPF-Net, FCN, and HSSAM-Net. The overlayed images further present how well the contours and shapes of such regions are captured by the HSSAM-Net model, which can already tell its superior handling for complex boundaries and small structure regions compared to the competing models. On the Kvasir dataset, which features highly variable polyp appearances, challenging lighting, and background textures, the segmentation outputs from UNet and Att-UNet reveal noticeable under-segmentation, where the predicted masks fail to fully capture the polyp boundaries. Seg-Net and DeepLabV3 demonstrate relatively better localization but tend to over-segment, often merging surrounding tissue into the polyp mask. CPF-Net and FCN exhibit inconsistent performance, particularly in the presence of small or low-contrast polyps, leading to false negatives and fragmented predictions. In contrast, HSSAM-Net produces segmentation maps that are both structurally coherent and spatially accurate, with sharp contours and complete coverage even in challenging regions. Its output overlays exhibit minimal deviation from the ground truth masks, indicating the model’s robust ability to differentiate foreground objects from background noise. In the CVC-ClinicDB dataset, which contains clearer frames with more distinct polyp structures, similar trends are observed. The SOTA models often fail to precisely delineate the polyp edges UNet and Seg-Net frequently leave border gaps, while DeepLabV3, despite improved performance, sometimes includes background noise within the mask. CPF-Net and FCN produce soft masks that do not consistently align with anatomical boundaries.

HSSAM-Net, on the other hand, consistently delivers precise segmentation, achieving high overlap with ground truth regions while maintaining well-preserved boundaries. The visual results reinforce the superiority of HSSAM-Net in capturing fine-grained polyp structures across datasets with varying complexity. The model’s design—particularly its Progressive Reusing Attention (PRA)**,** Max-Diagonal Pooling, and Hyper-Scale Aggregation modules enables it to learn both contextual semantics and spatial details effectively. Compared to SOTA methods such as UNet +  + , DeepLabV3, and C2FNet, HSSAM-Net consistently demonstrates clearer segmentation boundaries, fewer false positives, and greater fidelity to anatomical ground truth, confirming its robustness and generalization capabilities for real-world polyp segmentation tasks. The comparison clearly demonstrates that HSSAM-Net provides more accurate segmentation results, exhibiting fewer artifacts and a stronger alignment with ground truth annotations.

Figure 5 illustrates the qualitative segmentation results of our proposed HSSAM-Net compared to six widely-used SOTA models, evaluated across three datasets: ETIS, CVC-300, and EndoCV2020. For each dataset, the figure displays representative samples, with the first two columns showing the original endoscopic images and corresponding ground truth masks, columns 3–8 showing segmentation results from the baseline SOTA models, and the final column presenting the predicted mask generated by HSSAM-Net. This visualization highlights the effectiveness and robustness of HSSAM-Net under varying imaging conditions and polyp morphologies. On the ETIS dataset, known for its small image size, challenging illumination, and visually ambiguous polyp boundaries, the limitations of conventional models become evident. Models like UNet and Seg-Net tend to produce incomplete or fragmented segmentations, missing significant portions of the polyp regions. Att-UNet and FCN show moderate improvement but often fail to distinguish polyps from the surrounding mucosa, resulting in irregular and imprecise contours. DeepLabV3 and CPF-Net, which typically perform well on larger datasets, also exhibit false positives and under-segmentation, especially in low-contrast scenarios. In contrast, HSSAM-Net demonstrates superior delineation of polyp boundaries and better structural integrity, producing predictions that more closely resemble the ground truth across all test samples. Its robust feature aggregation and attention-guided decoding help it adapt to the noise and variability present in ETIS, ensuring improved generalization on smaller datasets.

Moving to the CVC-300 dataset, which consists of more uniform and well-centered polyp samples, several SOTA models including UNet, Seg-Net, and DeepLabV3—achieve reasonable segmentation quality. However, their outputs often lack smooth contours and precise boundary localization. Att-UNet and FCN frequently generate coarse masks that deviate from ground truth shapes, particularly around the periphery. CPF-Net slightly improves upon this by offering tighter boundary adherence but still fails to maintain consistency across samples. HSSAM-Net consistently generates highly accurate segmentation masks that are compact, well -aligned with ground truth, and devoid of spatial artifacts. These results underscore the effectiveness of our proposed modules in learning both local texture cues and global semantic context, even in cleaner and less ambiguous datasets. On the EndoCV2020 dataset, which poses a more diverse and clinically realistic segmentation challenge, the superiority of HSSAM-Net becomes even more apparent. Here, models like UNet, Att-UNet, and FCN often suffer from significant under-segmentation or fail to recognize the full extent of irregularly shaped polyps. While DeepLabV3 and CPF-Net provide better approximations, they still tend to either over-smooth the masks or leave out critical edge details. Notably, HSSAM-Net demonstrates excellent performance in both large and small lesion cases, capturing complex shapes and fine structures with remarkable precision. Its predictions show minimal discrepancy from the ground truth and exhibit a strong balance between segmentation tightness and coverage, validating the impact of progressive attention reusing, max-diagonal pooling, and hyper-scale shifted aggregation. These visual outcomes complement the quantitative metrics reported earlier and highlight HSSAM-Net’s potential for reliable and generalizable deployment in real-world clinical environments.

Table 5 presents the performance of the proposed HSSAM-Net across five widely used polyp segmentation datasets under various learning rates ranging from ${1\text{e}}^{-2}$ , ${1\text{e}}^{-3}, {1\text{e}}^{-4}$, ${2\text{e}}^{-3},$ ${2\text{e}}^{-4}$. Key segmentation metrics used for evaluation. This analysis investigates how the choice of learning rate affects model convergence, generalization, and structural segmentation accuracy. At a high learning rate ${1\text{e}}^{-2}$, HSSAM-Net demonstrates suboptimal performance across all datasets, with Dice scores ranging from 0.915 (ETIS) to 0.927 (CVC-ClinicDB), and relatively higher HD95 values (up to 19.20 on ETIS). This suggests unstable training and poorer boundary localization. In contrast, when the learning rate is reduced to ${1\text{e}}^{-3}$, segmentation results improve considerably Dice and mIoU increase across datasets, and HD95 drops, indicating better spatial alignment with ground truth masks. Notably, at ${1\text{e}}^{-4}$, HSSAM-Net achieves its best overall performance, with Dice scores of 0.953 on Kvasir**,** 0.956 on CVC-ClinicDB, and 0.955 on EndoCV2020. Corresponding HD95 values also improve substantially, reflecting highly precise and compact boundary predictions. These results confirm that HSSAM-Net benefits from a moderately low learning rate during optimization, allowing the network to fine-tune deep hierarchical features without overshooting or divergence. Interestingly, even at ${2\text{e}}^{-3}$ and ${2\text{e}}^{-4}$, HSSAM-Net maintains high accuracy, particularly with Dice scores above 0.94 across all datasets. Minor fluctuations in HD95 especially on ETIS and CVC-300 suggest that slightly lower learning rates may cause slower convergence or overfitting to train data in smaller datasets.

Among all configurations, the learning rate of ${2\text{e}}^{-4}$ emerges as the optimal choice, providing a balanced trade-off between segmentation overlap (Dice, mIoU) and spatial precision (HD95). Compared to existing SOTA models such as UNet +  + , C2FNet**,** and DeepLabV3, which typically achieve Dice scores in the range of 0.935–0.942 and mIoU values around 0.910 on most datasets, HSSAM-Net consistently surpasses these benchmarks under optimal learning conditions. For instance, on CVC-ClinicDB, DeepLabV3 reported a Dice of 0.935, while HSSAM-Net outperformed it with 0.956 Dice and a reduced HD95 of 12.12, confirming its enhanced ability to capture complex polyp structures with greater localization accuracy. Likewise, on the Kvasir dataset, where UNet +  + previously achieved a Dice of 0.940, HSSAM-Net yielded a superior result of 0.953 Dice at ${1\text{e}}^{-4}$, while simultaneously achieving the lowest HD95 (12.50). This consistently improved performance underscores the importance of selecting an appropriate learning rate and validates the architectural advantages introduced in HSSAM-Net.

### Ablation experiments

Ablation experiments were conducted to evaluate the progressive impact of architectural components integrated into HSSAM-Net in Table 6. Four variants (M₁–M₄) were designed to assess the contribution of each module, including MaxDP, MaxDUP, Local Attention Block (LAB), and PRA. These variants were evaluated on the CVC-ClinicDB and Kvasir datasets using Dice coefficient, HD95, mean Intersection over Union (mIoU), and Accuracy as evaluation metrics. The baseline variant M_1_, which excludes all enhancement modules, yields the lowest performance, with Dice scores of 0.895 and 0.920 on CVC-ClinicDB and Kvasir respectively, and relatively high HD95 values (21.30 and 18.80). This suggests limited capacity for fine boundary localization and contextual feature aggregation. Incorporating MaxDP and LAB in M₂ substantially boosts performance, especially in spatial consistency, improving Dice scores to 0.925 and 0.942 while lowering HD95 by approximately 3–4 points across both datasets. Further enhancement in M₃, through the addition of MaxDUP, results in notably stronger performance, particularly on the Kvasir dataset where it achieves the highest Dice (0.953), mIoU (0.940), and the lowest HD95 (12.50). On CVC-ClinicDB, M₃ also exhibits a clear gain with a Dice score of 0.954 and mIoU of 0.929. These findings underline the synergistic role of MaxDUP in improving decoder reconstruction quality when paired with MaxDP. The final full variant, M₄, integrates the full set of proposed modules and consistently delivers the best results across most metrics on CVC-ClinicDB—achieving a Dice score of 0.953, mIoU of 0.942, HD95 of 12.80, and the highest accuracy of 98.20%. On Kvasir, M₄ maintains competitive performance (Dice: 0.949, mIoU: 0.924, Accuracy: 98.72%), demonstrating that the inclusion of PRA not only enhances global context but also strengthens attention reusability in a multi-scale setting. The qualitative segmentation performance of four ablated variants of the proposed HSSAM-Net illustrated in Fig. 6. The visual results clearly validate the cumulative benefit of each proposed module and affirm the architectural design choices in HSSAM-Net.

Table 6 further expands the comparison by detailing the performance of HSSAM-Net and competing methods across all five datasets using the enriched set of evaluation metrics. This deeper analysis provides more nuanced insight into the strengths and weaknesses of each model. For instance, models such as DeepLabV3 and PraNet achieve competitive Dice and mIoU values, but their relatively higher MAE and lower TNR suggest reduced robustness against background false positives. In contrast, HSSAM-Net achieves the most balanced performance, consistently maintaining high Dice and mIoU while also demonstrating superior ACC, PPV, and TPR values across datasets. This balance indicates that HSSAM-Net not only captures polyp boundaries with high fidelity but also reduces misclassification errors, which is crucial in clinical applications where minimizing false positives and false negatives directly impacts diagnostic outcomes.

**Fig. 7:**
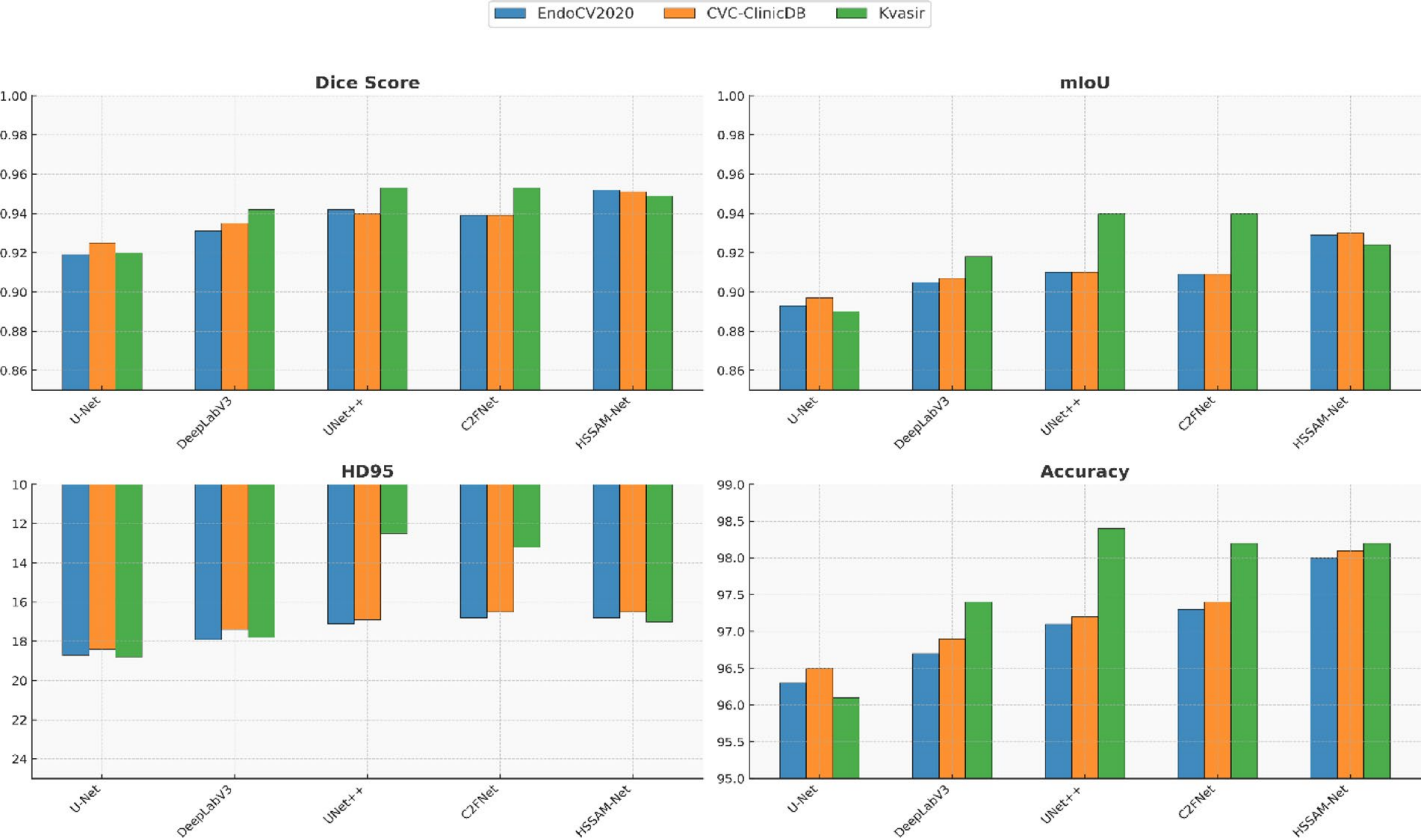
Five-fold cross-validation results (U-Net, DeepLabV3, UNet +  + , C2FNet, and HSSAM-Net) evaluated on EndoCV2020, CVC-ClinicDB, and Kvasir datasets. The bar plots compare performance across four key metrics. The HD95 metric is plotted with an inverted axis to visually emphasize lower values as better outcomes.

A five-fold cross-validation study was conducted to evaluate the generalization ability and segmentation performance of five widely used deep learning models U-Net, DeepLabV3, UNet +  + , C2FNet, and the proposed HSSAM-Net across three benchmark polyp segmentation datasets: EndoCV2020, CVC-ClinicDB, and Kvasir, shown in Fig. 7. The five-fold cross-validation results across EndoCV2020, CVC-ClinicDB, and Kvasir clearly demonstrate that HSSAM-Net offers superior performance in all major segmentation aspects region overlap, structural consistency, boundary accuracy, and classification reliability. These findings validate the architectural contributions of the model, particularly the PRA, MaxDP, and LAB mechanisms, and establish HSSAM-Net as a highly reliable solution for real-world clinical polyp segmentation tasks.

To evaluate the robustness and real-world applicability of HSSAM-Net, we conducted a cross-dataset generalization experiment shown in Table 7. This setting is particularly important because clinical datasets often differ in imaging conditions, resolutions, and annotation styles. The cross-dataset evaluation results are summarized, where models trained on CVC-ClinicDB were tested on unseen datasets (Kvasir and ETIS). The performance demonstrates the model’s ability to generalize beyond the training distribution. When tested on Kvasir, the model achieved a Dice score of 0.891 and mIoU of 0.870, with an HD95 of 21.4 and high accuracy (95.6%). Importantly, the PPV (0.897) and TPR (0.903) values indicate that the model maintains a good balance between precision and sensitivity in identifying polyps, while the TNR (0.965) reflects strong robustness against background false positives. On the more challenging ETIS dataset, the model understandably shows a slight decline, achieving a Dice score of 0.868 and mIoU of 0.845, with HD95 increasing to 24.8. Nonetheless, the accuracy (94.2%) remains high, and the PPV (0.902) and TPR (0.909) confirm that the model continues to reliably detect polyps despite domain shifts. These results suggest that the proposed HSSAM-Net demonstrates strong cross-dataset generalization, preserving accuracy and precision even when tested on datasets with significant differences in imaging conditions and annotation characteristics.

**Table 7:** Cross-dataset generalization results of HSSAM-Net.

Training dataset	Test dataset	Dice score	mIoU	HD95	Accuracy	MAE	PPV	TPR	TNR
CVC-ClinicDB	Kvasir	0.891	0.870	21.4	95.6	3.9	0.897	0.903	0.965
CVC-ClinicDB	ETIS	0.868	0.845	24.8	94.2	3.8	0.902	0.909	0.967

The computational efficiency and processing speed between the proposed HSSAM-Net model and SOTA models for image segmentation presented in Table 8. The comparison is done with respect to three important key indicators: FLOPs, the number of model parameters in millions, and frames per second (FPS), which reflects the processing speed. The results indicate that HSSAM-Net has better computational efficiency, with a FLOP count of only 4.3 billion, outperforming huge models like UNet +  + at 251.2 billion FLOPs and U-Net at 118.1 billion FLOPs by a large margin. This huge reduction of computation will automatically lead to less power consumption and reduced processing time; hence, HSSAM-Net is quite useful for resource-constrained deployments. To further demonstrate the stability and reliability of the training process, we analyzed the convergence behavior of HSSAM-Net on the Kvasir and CVC-ClinicDB datasets. The training and validation curves of Dice coefficient and loss showed smooth convergence with minimal gaps, indicating robust generalization without signs of overfitting or divergence. For clarity, the detailed learning curves are provided in the Supplementary Material (SI Fig. 1).

**Table 8:** The comparison of HSSAM-Net with state of the art models in terms of FPS, FLOPs and parameters.

Models	FLOPs(G)	Parameters (M)	Speed (FPS)
UNet^64^	118.1	33.5	118.5
Seg-Net^65^	12.7	36.2	45.5
DeepLabV3^66^	16	24.5	20.9
FCN^67^	18.5	28.1	30.6
MSNet^68^	10.0	25.2	19.5
Att-U Net^69^	13.6	34.2	15.3
CFA-Net^70^	53.3	26.5	23.5
UNet + +^66^	251.2	30.2	74.1
C2FNet^71^	15.7	35.3	65.3
PraNet^66^	17.5	14.3	25.5
SFA^72^	14.1	19.5	38.4
Proposed HSSAM-Net	4.3	0.9	24.1

Besides that, the model size of HSSAM-Net is very small, only 0.9 million parameters. Compared to other architectures, for example, C2FNet-35.3 million and SegNet-36.2 million, the parameters of this proposed method are comparatively low. It leads to faster training with reduced memory requirements, increasing the suitability for resource-limited environments. It is impressive to note that despite the drastically reduced computational complexity and much smaller model size, the achieved competitive processing speed for HSSAM-Net is 24.1 FPS. It is rather decent compared with DeepLabV3’s 20.9 FPS and MSNet’s 19.5 FPS, thus suggesting that its efficiency gain does not come at the cost of real-time performance.

The HSSAM-Net dynamically adjusts the reception field, retaining the salient fine-grained features in both the downsampling and upsampling processes; thus, this brings significantly higher performance than traditionally designed U-Net Variants, which usually suffer due to the loss of the fine spatial details caused either by fixed-size receptive fields or by excessive loss from resampling. Not introducing redundancy while aggregating the features across different scales enriches the capability of a segmentation model to precisely catch large and small-size structures. This point especially becomes very important within the polyp segmentation context where size, shape, and contrast for a polyp may change fairly prominently. Equally important, the proposed Max-Diagonal Sampling allows more focused sampling concerning image features, being much more attentive to the most informative pixels with the view to enhancing segmentation accuracy. In this way, it allows the model to focus on areas of interest that, in the majority of cases, are underrepresented by classic uniform sampling. This helps in strengthening the robustness of the model, especially for images where polyps could appear in low-contrast or irregular regions.

### Implications, challenges, and future research directions

The proposed HSSAM-Net has several important implications for clinical and real-world applications. First, by achieving accurate and real-time segmentation of colorectal polyps, the model can serve as a decision support tool for gastroenterologists during colonoscopy, thereby reducing miss rates and facilitating early detection of colorectal cancer. Second, owing to its lightweight design with only ~ 0.9 M parameters and low FLOPs, HSSAM-Net can be efficiently deployed in resource-constrained environments, including portable colonoscopy systems or embedded medical devices, broadening access to advanced diagnostic support. Third, the consistent and objective delineation of polyps across diverse datasets helps reduce inter-observer variability, contributing to more standardized diagnosis and documentation in clinical practice. Finally, the model’s robustness and efficiency make it suitable for integration into computer-aided detection and diagnosis (CADe/CADx) systems, providing real-time feedback to clinicians and enhancing workflow efficiency. Together, these practical implications underscore the clinical relevance of HSSAM-Net and its potential to support reliable, efficient, and widely deployable colorectal cancer screening solutions.

Despite its strong performance, several challenges remain. First, although the model generalizes well across multiple public datasets, its robustness under domain shifts caused by varying camera hardware, patient demographics, or procedural protocols in real-world clinical settings has yet to be comprehensively tested. Second, the model’s performance on extremely small or heavily occluded polyps cases that are particularly challenging and clinically significant could benefit from further enhancement. Additionally, the inference time and computational cost, while moderate, may still pose barriers to deployment on low-resource or real-time embedded systems without further optimization.

Looking ahead, several future research directions emerge. One promising avenue is the integration of semi-supervised or self-supervised learning, which can leverage large amounts of unlabeled endoscopic data to improve model generalization without reliance on extensive manual annotation. Incorporating temporal information from video frames rather than processing single frames independently may also improve detection consistency and reduce false positives in live procedures. Moreover, multi-modal fusion combining RGB images with depth, texture, or spectral data could further enhance boundary delineation in difficult cases. From a deployment perspective, efforts should be directed toward developing lightweight or quantized variants of HSSAM-Net for real-time use in resource-constrained environments such as portable colonoscopy units or mobile diagnostic platforms.

## Conclusion

In this study, we proposed HSSAM-Net, a novel and lightweight U-Net-based architecture tailored for precise colorectal polyp segmentation in endoscopic images. The model integrates several key innovations, including the Hyper-Scale Shifted Aggregation Module for enriched multi-scale feature fusion, the Progressive Reusing Attention (PRA) mechanism for enhanced feature reusability, and the dual-branch Max-Diagonal Pooling/Unpooling (MaxDP/MaxDUP) strategy for improved geometric consistency. These components work synergistically to overcome common limitations of existing segmentation methods, such as loss of fine-grained details, inefficient feature aggregation, and high computational cost. Extensive experiments across five benchmark datasets (Kvasir, CVC-ClinicDB, ETIS, CVC-300, and EndoCV2020) demonstrated that HSSAM-Net consistently outperforms state-of-the-art models in both region-based and boundary-aware metrics. Notably, the network achieved superior Dice and mIoU scores while maintaining an extremely low parameter count (~ 0.9 M) and real-time inference speed (24.1 FPS). These results confirm that HSSAM-Net achieves an effective balance between segmentation accuracy and computational efficiency, making it suitable for integration into real-time computer-aided diagnosis systems. Beyond quantitative performance, qualitative evaluations highlighted the model’s robustness in capturing small, low-contrast, and irregularly shaped polyps, underscoring its potential clinical utility. The lightweight design also makes it adaptable for deployment in resource-constrained settings, such as portable colonoscopy systems or embedded medical devices, broadening access to advanced diagnostic support. At the same time, we acknowledge certain limitations of the present work, including the need for further validation under real-world domain shifts, challenges in extremely diminutive or occluded polyp cases, and the requirement of additional optimization for ultra-low-power embedded deployment. Future work will therefore focus on addressing these limitations by exploring semi-supervised or self-supervised learning, multi-modal data fusion, and prospective validation on multi-center clinical datasets. HSSAM-Net provides a powerful, efficient, and clinically relevant solution for automated colorectal polyp segmentation, advancing the state of the art while laying the groundwork for broader clinical translation.

## Supplementary Information

Below is the link to the electronic supplementary material.

Supplementary Material 1

## Data Availability

The datasets used/analysed in our study are publicly available (https://polyp.grand-challenge.org/CVCClinicDB/; https://datasets.simula.no/kvasir-seg/; http://mv.cvc.uab.es/projects/colon-qa/cvc-colondb; http://adas.cvc.uab.es/endoscene).
