# Supplementary material for: HSSAM-Net: hyper-scale shifted aggregation network for precise colorectal polyp segmentation in endoscopic images
Source: Sci Rep. 2025 Oct 31;15:38146. doi: 10.1038/s41598-025-21954-y (PMC12578911; doi:10.1038/s41598-025-21954-y)
Supplement: Supplementary file 1 — Supplementary Material 1 [file 41598_2025_21954_MOESM1_ESM.docx]

Supplementary Materials


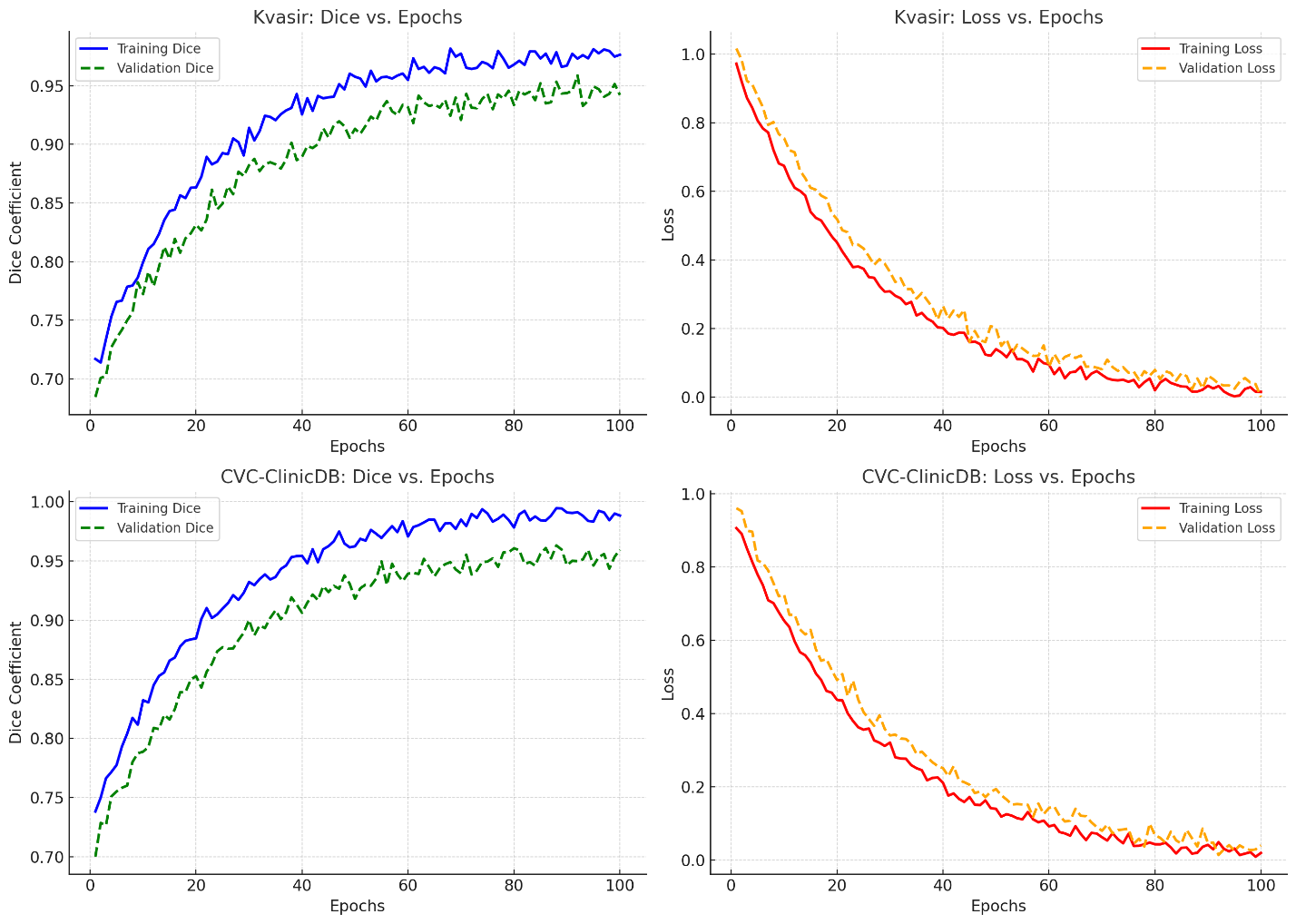


SI Figure 1. Training and validation learning curves of HSSAM-Net on two benchmark datasets. (a) Kvasir: Dice coefficient vs. epochs. (b) Kvasir: Loss vs. epochs. (c) CVC-ClinicDB: Dice coefficient vs. epochs. (d) CVC-ClinicDB: Loss vs. epochs. The curves show smooth convergence with minimal gap between training and validation, indicating reliable optimization and strong generalization.
